# Endovascular management of hemosuccus pancreaticus, a rare case report of gastrointestinal bleeding

**DOI:** 10.1186/s12876-016-0418-3

**Published:** 2016-01-14

**Authors:** Hye Ryoung Sul, Hyun Woong Lee, Jeong Wook Kim, Sung Jae Cha, Yoo Shin Choi, Gi Hyeon Kim, Byung Kook Kwak

**Affiliations:** Department of Internal Medicine, Chung-Ang University College of Medicine, 224-1 Heuk Seok-Dong, Dongjak-Ku, Seoul 156-755 Republic of Korea; Department of Surgery, Chung-Ang University College of Medicine, Seoul, Republic of Korea; Department of Radiology, Chung-Ang University College of Medicine, Seoul, Republic of Korea

**Keywords:** Hemosuccus pancreaticus, Aneurysm, Splenic artery, Stent

## Abstract

**Background:**

Hemorrhage from the pancreatic duct, or hemosuccus pancreaticus (HP), is an unusual cause of intermittent gastrointestinal bleeding. HP is most often diagnosed in patients with chronic pancreatitis, and is usually due to the rupture of an aneurysm in the splenic artery. The traditional treatment for HP is surgery, although most cases can be managed by angioembolization.

**Case Presentation:**

We present a case of HP in a patient with no history or evidence of chronic pancreatitis. Repeated endoscopy revealed fresh bleeding from the papilla of Vater. Angiography revealed an aneurysm of the splenic artery, which was the suspected cause of the intermittent bleeding from the pancreatic duct. Angiography demonstrated extravasation of contrast from the aneurysm. A peripheral Jostent stent-graft was hand-mounted on an angioplasty balloon and then inserted into the aneurysm. Arteriography revealed successful occlusion of the aneurysm with the stent-graft. No recurrent gastrointestinal bleeding was observed during the five years follow-up periods.

**Conclusion:**

HP should be included in the differential diagnosis of intermittent gastrointestinal bleeding in patients with histories of chronic alcoholism, even when they do not have a history of chronic pancreatitis. We recommend an interventional procedure with a metal stent for the initial treatment of HP.

## Background

Several terms have been used to describe hemorrhage from the pancreatic duct, including wirsumgorrhagia, proposed by Van Kemmel in 1969, the common terminology used in France, or the equivalent hemowirsungia. Hemosuccus pancreaticus (HP) was proposed by Sandblom in 1970, and hemoductal pancreatitis by Longmire and Rose in 1973. All of these terms describe the emission of red blood along the pancreatic duct through the papilla. Mentions of HP in the medical literature remain mostly limited to case reports [1, 2]. Hemorrhage from abdominal vessels other than the splenic artery, such as the hepatic artery, leading to HP is rare [3]. Aneurysm and chronic pancreatitis are often associated, but no causal relationship has been established. Local inflammation with or without a concomitant pseudocyst, possibly combined with local release of elastase or even pressure necrosis from ductal calculi, induce pseudocyst formation. Other pancreatic causes of HP are rare and include pancreatolithiasis, ectopic pancreas, and pancreas divisum [4, 5].

HP caused by arterial aneurysm is conventionally treated surgically, although interventional radiological procedures are now being utilized. However, implantation of a metal stent may be an effective procedure for the treatment of HP. We report on a case of a peripheral Jostent stent-graft insertion for the management of a HP.

## Case Presentation

A 48-year-old male was referred to our institution with a primary complaint of six months of intermittent relapsing melena of unknown origin. He had no additional symptoms and did not have a history of abdominal surgery. Although the patient was a chronic alcoholic, he did not have a history of pancreatitis. Several times of upper and lower endoscopies revealed melena with multiple diverticulae in the ascending colon, but failed to identify a source of the relapsing melena.

**Fig. 1:**
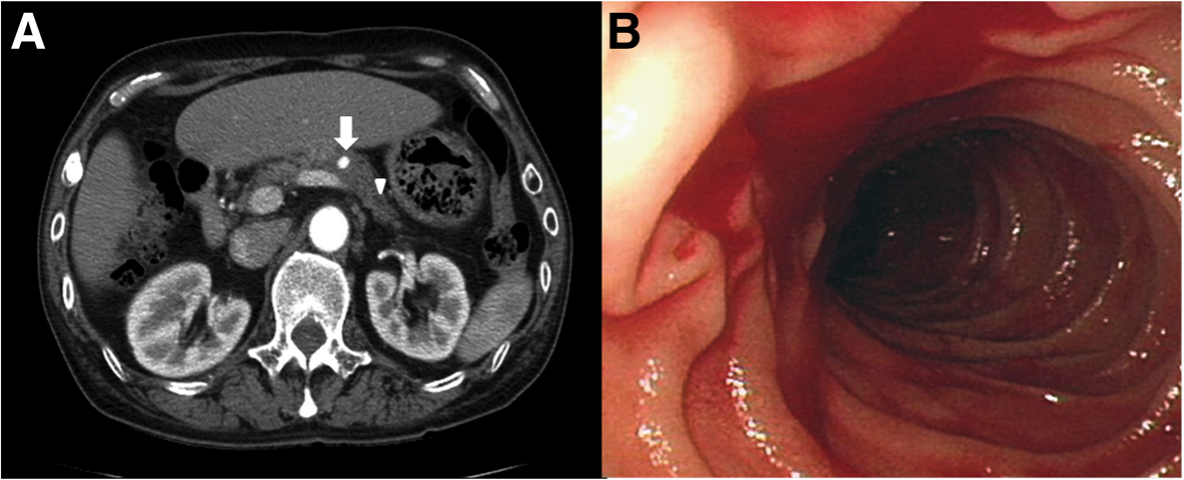
Abdominal CT scan and Endoscopic findings. **a** 6.0 mm × 10.4 mm saccular aneurysm was evident in the splenic artery (pancreatic segment of the splenic artery) at the body-tail junction of the pancreas (arrow). The aneurysm was out-pouching from the splenic artery perpendicularly down to the pancreas parenchyma. The aneurysm contacted the pancreatic duct, and the pancreatic duct proximal to the aneurysm was not dilated. However, the distal pancreatic duct was slightly dilated (up to 4.0 mm) (arrow head). The surrounding pancreas, especially distal to the aneurysm, was slightly lower in density after contrast enhancement. This finding suggests swelling. **b** On admission day 9, esophagogastroduodenoscopy showed active hemorrhage from the major papilla

On examination, the patient exhibited pallor, his blood pressure was 120/70 mmHg, and pulse was 78/min. His abdominal examination revealed no pathological findings, except for black, tarry stool on digital rectal examination. Laboratory studies revealed a normocytic normochromic anemia (hemoglobin 9.8 mg/dl, hematocrit 30.1 %, mean corpuscular volume 89.9 fl, mean corpuscular hemoglobin 29.3 pg). Folate and vitamin B12 levels were normal, and the peripheral blood smear was compatible with chronic blood loss. Other laboratory tests were normal. Upper abdominal ultrasonography did not show any pathological findings. On abdominal computerized tomography (CT), a 6.0 mm × 10.4 mm saccular aneurysm was evident in the pancreatic segment of the splenic artery at the body-tail junction of pancreas. The aneurysm was out-pouching from the splenic artery perpendicularly down to the pancreas parenchyma, and the aneurysm contacted the pancreatic duct. The pancreatic duct proximal to the aneurysm was not dilated; however, the distal pancreatic duct was slightly dilated up to 4.0 mm (Fig. 1a). There was no extravasation of contrast media into the pancreatic duct, pancreatic parenchyma, or the bowel lumen. The surrounding pancreas, especially distal to the aneurysm, was slightly lower in density after the administration of contrast, probably due to swelling. Additionally, there was no parenchymal calcification, pancreaticolith, or peripancreatic fluid collection on CT.

On the ninth day after admission, the patient experienced sudden hematemesis and melena. An emergency esophagogastroduodenoscopy (EGD) was performed and revealed fresh blood in the stomach and duodenum with active hemorrhage from the major papilla, a finding suggestive of hemosuccus pancreaticus (Fig. 1b). Follow-up abdominal CT showed that the aneurysm was slightly increased in size (6.3 mm × 11.0 mm). Otherwise, there were no interval changes in the pancreatic duct dilatation and lower pancreatic enhancement. No extravasation of contrast media was observed.

**Fig. 2:**
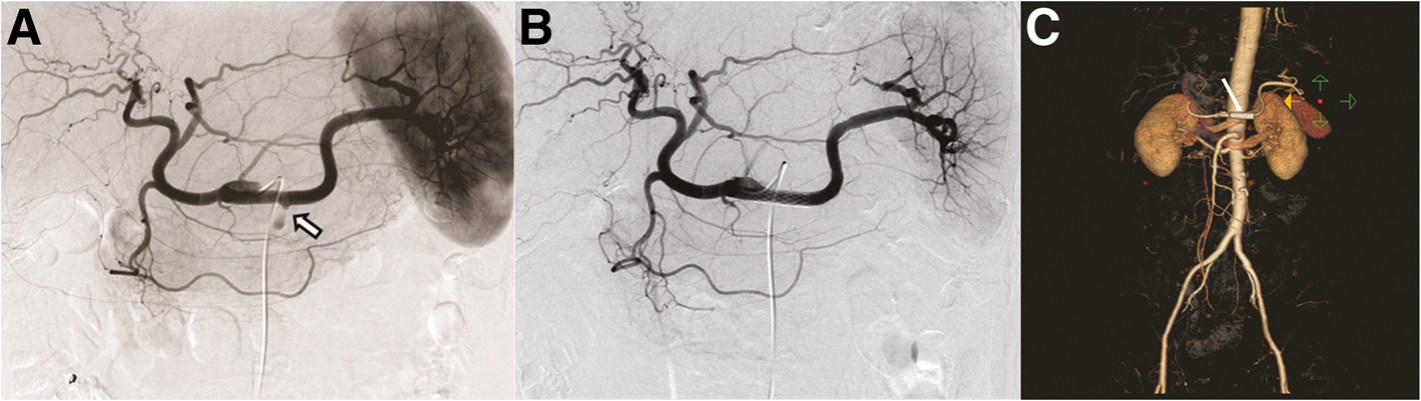
Angiographic finding and endovascular treatment. **a** Angiography of the splenic artery revealed a 5-mm saccular aneurysm of the splenic artery (arrow). **b** The splenic artery aneurysm after implantation of a 28-mm Jostent. **c** On a contrast-enhanced abdominal angiography CT scan obtained 10 days after the procedure, the splenic artery aneurysm was completely excluded (Stent, arrow)

Because the EGD findings were suggestive of hemosuccus pancreaticus (HP), angiography of the celiac artery as well as the upper and lower mesenteric arteries was performed. Under local anesthesia and conscious sedation, by catheterization through the right groin, a catheter was positioned in the proximal part of the abdominal aorta. Angiography confirmed the splenic artery aneurysm (SAA) in the pancreatic segment (Fig. 2a). An 8-F Mach 1 guide catheter (Boston Scientific, Natick, MA, USA) was inserted over a stiff-type, 0.035-in., 260-cm Radifocus Terumo guidewire (Terumo, Tokyo, Japan) up to the proximal splenic artery without an arterial sheath. A 4- to 9-mm-diameter and 28-mm-long peripheral Jostent stent-graft (Jostent Graftmaster, Abbott, Rangendingen, Germany) was hand-mounted on a 6-mm-wide and 4-cm-long angioplasty balloon (Ultra-Thin Diamond, Boston Scientific, Watertown, MA, USA). Through the 8-F Mach 1 guide catheter over the stiff-type, 0.035-in., 260-cm Radifocus Terumo guidewire (Terumo), the stent-graft was inserted to the SAA and deployed by inflating the balloon. Arteriography performed immediately after the procedure revealed successful occlusion of the aneurysm with the stent-graft (Fig. 2b).

On a contrast-enhanced abdominal angiography CT scan obtained 10 days after the procedure, the SAA was completely excluded, but the affected artery and spleen were preserved (Fig. 2c). Additionally, the patient’s gastrointestinal bleeding was improved. At the time of clinical follow-up four year after the procedure, the patient reported no further episodes of bleeding, and his hemoglobin levels remained stable.

## Discussion

The key symptom of HP is melena. Hematemesis is less frequent, and rupture into the abdominal cavity or the retroperitoneum is rare [6]. It is difficult to diagnose HP because the bleeding is usually intermittent. An endoscopic examination of the upper gastrointestinal tract may reveal bleeding from the pancreatic duct. EGD can also show normal findings, and other causes of upper digestive bleeding (erosive gastritis, peptic ulcer, esophageal, and gastric fundus varices) should be ruled out. While the coexistence of chronic, calcifying pancreatitis with HP is the norm, this case report shows that HP is possible in a patient without a history of pancreatitis. Although our patient did not have chronic pancreatitis, he was a chronic alcoholic, and his pancreas may have shown histological signs suggestive of chronic pancreatitis if biopsied. Thus, pancreatic enzyme may erode pancreatic parenchyma and cross tissue boundaries, resulting in connection with adjacent aneurysm. Other uncommon causes of the splenic arterial aneurysm are fibromuscular dysplasia, segmental arterial mediolysis and systemic vasculitis. However, there were no clinical or laboratory findings suggestive of vascular disease.

Once the patient is hemodynamically stable, interventional procedures are effective as an initial treatment in 67 % to 100 % of cases [7]. However, embolization procedures, in which the splenic artery is embolized by platinum spirals or gel foam, may lead to splenic infarction. Coil embolization techniques provoke a thrombus in the aneurysm but also obliterate the artery [6]. Ischemia can develop in the tissue supplied by the artery if the collateral circulation is not sufficient. Embolization of the celiac trunk, the common hepatic artery, or the superior mesenteric artery is thus contraindicated. Yet another possible complication of these procedures is aneurysm infection. Although the embolization of splenic artery is also effective technology, spleen abscess or septic complication would be developed.

Benz et al. recently used an interventional procedure to treat HP, in which an uncoated metal Palmaz stent was placed across the aneurysmic segment of the splenic artery [8]. This report suggests that implantation of a metal stent may be an effective treatment for HP with low rates of recurrence and complications. Nevertheless, stent graft of aneurysms should be avoided in these cases. (1) vessel tortuosity, (2) small caliber size, (3) proximal and distal neck size mismatch. In other words, accessibility to stent grafts in an emergency situation may not be possible. The alternate modes of treatment other than surgery (e.g. EUS guided thrombin injection or coiling of the segment of splenic artery or use of glue to fill the sac of pseudoaneurysm) would be also helpful.

Surgical treatment is indicated in uncontrolled hemorrhage, persistent shock, and when embolization is not feasible. Surgical procedures consist of resection, laparoscopic in part, or ligature of bleeding vessels [9]. Most surgical series have documented a success rate of 70 % to 85 %, with mortality rates of 20 % to 25 %, and rebleeding rates of 0 % to 5 % [10, 11].

This case report suggests that even if a patient does not have any evidence of chronic pancreatitis, HP must be included in the differential diagnosis for chronic alcoholics with intermittent upper gastrointestinal bleeding. In this patient, the treatment was successful without complication or rebleeding for five years after therapy.

## Conclusion

Repeated examinations and careful observation should be performed to find obscure sources of repeated upper gastrointestinal bleeding. HP should be included in the differential diagnosis of intermittent gastrointestinal bleeding in patients with histories of chronic alcoholism, even when they do not have a history of chronic pancreatitis. Moreover, as HP may be caused by small aneurysms, it is important to utilize imaging that affords appropriate diagnostic accuracy when trying to rule out HP. We recommend an interventional procedure for the initial treatment of HP, and feel that surgical treatment should only be considered when the patient is unstable, when angiography shows no abnormal findings, or when the interventional therapy is not successful. Furthermore, implantation of a metal stent appears to be an effective treatment for HP.

### Consent

Written informed consent was obtained from the patient for publication of this Case report and any accompanying images. A copy of the written consent is available for review by the Editor of this journal.

## Abbreviations

HP: Hemosuccus pancreaticus
CT: Computerized tomography
EGD: Esophagogastroduodenoscopy
SAA: Splenic artery aneurysm

## References

[1] Etienne S, Pessaux P, Tuech JJ, Lada P, Lermite E, Brehant O (2005). Hemosuccus pancreaticus: a rare cause of gastrointestinal bleeding. Gastroenterol Clin Biol.

[2] Panackel C, Kumar A, Subhalal N, Krishnadas D, Kumar KR. Education and imaging. Hepatobiliary and pancreatic: hemosuccus pancreaticus complicating calcific chronic pancreatitis. J Gastroenterol Hepatol. 2007;22(10):1691. 10.1111/j.1440-1746.2007.05146.x 17845697

[3] De Mas R, Kohler B, Ante D, Schonleben K, Riemann JF (1989). Hemosuccus pancreaticus following rupture of a hepatic artery aneurysm. Z Gastroenterol.

[4] Jakobs R, Riemann JF (1992). Hemosuccus pancreaticus due to a pressure ulcer in pancreatolithiasis. Dtsch Med Wochenschr.

[5] Meneu JA, Fernandez-Cebrian JM, Alvarez-Baleriola I, Barrasa A, Morales V, Carda P (1999). Hemosuccus pancreaticus in a heterotopic jejunal pancreas. Hepatogastroenterology.

[6] Kuhn R, Janocha F, Lazar A, Rambach W, Paquet KJ (1996). Ruptured pseudoaneurysm of the splenic artery. A complication of chronic pancreatitis. Dtsch Med Wochenschr.

[7] Gambiez LP, Ernst OJ, Merlier OA, Porte HL, Chambon JP, Quandalle PA (1997). Arterial embolization for bleeding pseudocysts complicating chronic pancreatitis. Arch Surg.

[8] Benz CA, Jakob P, Jakobs R, Riemann JF (2000). Hemosuccus pancreaticus--a rare cause of gastrointestinal bleeding: diagnosis and interventional radiological therapy. Endoscopy.

[9] Saw EC, Ku W, Ramachandra S (1993). Laparoscopic resection of a splenic artery aneurysm. J Laparoendosc Surg.

[10] Bender JS, Bouwman DL, Levison MA, Weaver DW (1995). Pseudocysts and pseudoaneurysms: surgical strategy. Pancreas.

[11] Heath DI, Reid AW, Murray WR (1992). Bleeding pseudocysts and pseudoaneurysms in chronic pancreatitis. Br J Surg.

